# Identification of ubiquitination-related signature genes for predicting kidney transplant rejection

**DOI:** 10.1038/s41598-026-38022-8

**Published:** 2026-02-10

**Authors:** Zhengfei Shan, Shengqiang Yu, Jiantao Wang, Jianxin Cui, Haijian Wei, Xiaohua Fu, Chen Zhang, Chengjun Zhang

**Affiliations:** Department of Organ Transplantation, Yantai Yuhuangding Hospital, No. 20 East Yuhuangding Road, Yantai, 264000 Shandong China

**Keywords:** Kidney transplantation, Transplant rejection, Gene expression signatures, Ubiquitination, Immune infiltration, Neutrophil cytosolic factor 4, Biomarkers, Computational biology and bioinformatics, Diseases, Genetics, Immunology, Nephrology

## Abstract

Kidney transplant rejection (KTR) poses significant challenges to long-term graft survival, with involvement from ubiquitination-related genes (URGs) in immune modulation. This study aimed to identify key URGs linked to KTR and develop a predictive model for rejection risk. mRNA array data from the Gene Expression Omnibus were analyzed to find differentially expressed genes in GSE98320, which were intersected with URGs to yield 16 DE-URGs. Gene Ontology and KEGG enrichment analysis highlighted the NF-kappa B and TNF signaling pathways. A URGScore model stratified patients and revealed significant differences in immune cell infiltration, especially among Treg cells, demonstrating strong predictive performance in the discovery cohort (AUC = 0.774, 95% CI 0.747–0.800). Six signature genes (*DTX3L*,* MARCH1*,* NCF4*,* RNF125*,* TRIM21*,* TRIM22*) were identified, and their expression displayed a subtype-dependent gradient, increasing from antibody-mediated rejection to T cell-mediated rejection and reaching the highest levels in mixed rejection. These genes were incorporated into a nomogram, which achieved an AUC of 0.771 (95% CI 0.745–0.798). Validation in independent datasets confirmed the model’s reliability. In the two transplant rejection cases, *MARCH1* and *RNF125* showed higher expression than the other biopsy samples, while generalized high expression of all marker genes was observed in an IgA nephropathy patient. Together, these findings demonstrate the clinical relevance of URG-based biomarkers in KTR and provide molecular insight into immune-mediated rejection.

**Supplementary Information:**

The online version contains supplementary material available at 10.1038/s41598-026-38022-8.

## Introduction

Kidney transplantation is the optimal treatment for improving survival and quality of life in patients with end-stage kidney disease^1^. However, rejection occurs when the recipient’s immune system recognizes the donor kidney as foreign and attacks it, which is typically classified into T-cell-mediated rejection (TCMR) and antibody-mediated rejection (ABMR)^2^. In TCMR, T-lymphocytes trigger tubulointerstitial inflammation, while in ABMR, antibodies target the donor’s human leukocyte antigens (HLA), leading to microvascular inflammation^3^. In clinical practice, distinguishing these categories is considerably complex, as renal allograft biopsies often display overlapping lesions. Microvascular inflammation, tubulitis, interstitial inflammation, peritubular capillaritis, and variable C4d deposition frequently coexist, and many cases fulfill criteria for mixed rejection or fall into borderline or indeterminate patterns^4^. These overlaps reflect the combined contributions of humoral and cellular immunity to graft injury, making binary classification challenging in routine pathology.

Within the first year post-transplant, approximately 10–15% of patients experience rejection^5^, driven by complex interactions of innate and adaptive immune responses. Kidney transplant rejection (KTR) may cause allograft dysfunction, graft failure, and increased morbidity^6^. Current treatment strategies primarily involve immunosuppressive therapies to prevent or mitigate rejection episodes^7^. However, rejection episodes can still occur, and the clinical management of transplant recipients remains challenging due to the limitations of current diagnostic tools. For example, serum creatinine, commonly used to monitor kidney function, detects rejection only after significant injury, while renal biopsies, the gold standard, are invasive and carry risks of complications, limiting their frequent use^8^. Identifying reliable biomarkers that can detect early signs of rejection and reduce the need for tissue biopsies is essential for improving long-term outcomes in kidney transplant patients.

Ubiquitination is a post-translational modification in which ubiquitin molecules are covalently attached to substrate proteins, thereby regulating their stability, localization, and signaling activity^9^. In immune responses, ubiquitination critically controls antigen presentation, T-cell activation, and cytokine signaling^10^. Ubiquitination plays a key role in KTR by regulating immune responses and inflammation, particularly through the activity of ubiquitin ligases that are associated with T-cell activation and the degradation of immune-modulating proteins^11^. Elevated levels of proteins involved in ubiquitination, such as small ubiquitin-related modifier 2 (SUMO2) and F-box protein FBXL19, have been observed in KTR^12^, targeting damaged or misfolded proteins for degradation^13^. This process influences key immune pathways, including antigen presentation, T-cell activation, and inflammatory signaling, which exacerbates ischemia-reperfusion injury (IRI) and contributes to chronic allograft dysfunction^14^. Moreover, components of the ubiquitin–proteasome system are dysregulated in both acute and chronic rejection^15,16^, and E3 ligases such as Cbl-b and Itch play essential roles in maintaining T cell anergy and immune tolerance^17^. Given its position as an upstream regulator of multiple immune signaling cascades, perturbations in ubiquitination may emerge earlier and exert broader effects than downstream inflammatory markers, providing an opportunity to identify earlier and more mechanistically informative biomarkers in KTR. Although ubiquitination-related proteins have been proposed as biomarkers in liver transplantation^15^, no studies have identified ubiquitination-related genes (URGs) as biomarkers in KTR, leaving this area largely unexplored. This is likely because its regulatory network is highly complex, involving interconnected E1, E2, and E3 enzymes and context-dependent substrate specificity, making traditional candidate-based biomarker discovery difficult. By contrast, transcriptome-wide analyses and integrative machine-learning approaches, as used in this study, are well suited to capture coordinated changes across multilayered pathways and can identify potential signatures that might not be detectable through single-gene or hypothesis-driven approaches.

This study aimed to develop a diagnostic model for KTR based on URGs by analyzing multiple transcriptomic datasets. We constructed and validated a URGScore model to quantify ubiquitination-related activity, identify signature URGs associated with KTR, and further validated the expression of these key genes in our own cohort of patient biopsy samples. Our findings demonstrated the effectiveness of the URGScore in predicting rejection risk and highlighted key URGs as potential biomarkers for KTR.

## Materials and methods

### Study cohorts and data sources

The mRNA array data were obtained from the Gene Expression Omnibus database (GEO) database (https://www.ncbi.nlm.nih.gov/geo/), including GSE98320 (1,208 kidney transplant biopsies: 692 no rejection, 109 boderline, 279 ABMR-related, 87 TCMR, 41 mixed)^18^, GSE48581 (300 kidney samples: 32 TCMR, 40 ABMR, 6 mixed rejection, 46 borderline, 20 transplant glomerulopathy, 13 polyomavirus nephropathy, 40 glomerulonephritis, 14 acute kidney injury, 43 no major abnormalities, 38 interstitial fibrosis/tubular atrophy, and 8 other diagnoses)^19^, and GSE50058 (101 allograft biopsy samples from multiple organs, including 43 acute rejection and 58 non-rejection, without organ-specific subtype annotations)^20^. In both GSE98320 and GSE48581, borderline lesions were classified as non-rejection for binary rejection versus non-rejection analyses. The sample details are summarized in Table 1. GSE98320 was used as the discovery dataset, while GSE48581 and GSE50058 served as validation datasets. Raw CEL files were downloaded and normalized using the RMA function from the “affy” package. Probes were mapped to gene symbols, with empty probes removed, and for genes with multiple probes, the mean probe value was used. The ComBat function from the “sva” package was applied to correct for batch effects across datasets. The dataset ID was specified as the batch variable to account for platform and study-specific variation. Biological covariates were not included in the model due to inconsistencies in phenotype annotations across datasets. This approach allowed harmonization of gene expression data while minimizing the risk of overfitting or introducing bias from incomplete clinical labels. A total of 806 ubiquitination-related genes (URGs) were sourced from a published study^21^.

**Table 1:** Study cohorts and data sources.

ID	Platform	Sample type	Sample size (rejection: non-rejection)	Rejection subtype distribution	Data type
GSE98320	GPL15207	Kidney tissue	1208 (407:801)	ABMR-related (279); TCMR (87); Mixed (41)	mRNA array
GSE48581	GPL570	Kidney tissue	300 (78:222)	TCMR (32); ABMR (40); Mixed (6)	mRNA array
GSE50058	GPL570	Multi-organ allograft biopsies	101	AR (43); No rejection (58); Subtype data not available	mRNA array

*ABMR* antibody-mediated rejection, *TCMR* T cell-mediated rejection, *Mixed* combined ABMR and TCMR, *AR* acute rejection.

### Differential expression analysis

Differential gene expression analysis was conducted on the GSE98320 dataset to compare KTR samples with control groups using the “limma” R package (version 3.50.3). Multiple testing was corrected by the Benjamini–Hochberg method. DEGs were identified with thresholds of |logFC| > 0.3^22,23^ and adjusted *p* value < 0.05. These DEGs were then intersected with URGs, and the overlapping genes were defined as DE-URGs.

### Functional enrichment analysis

Gene Ontology (GO) and Kyoto Encyclopedia of Genes and Genomes (KEGG)^24–26^ pathway analyses of the DE-URGs were performed using the “clusterProfiler” R package (version 4.2.2)^27^. The enrichment results were visualized using the “enrichment plot” R package (version 1.14.1).

### Construction and validation of the URGScore model

The URGScore model was constructed to quantify ubiquitination-related activity using DE-URGs. The URGScore for each sample was calculated via single-sample gene set enrichment analysis (ssGSEA) using the “GSVA” package (version 1.42.0). Patients were then categorized into low and high URGScore groups based on the median ssGSEA score. Principal component analysis (PCA) was applied to verify the expression heterogeneity of DE-URGs between these subgroups. The predictive accuracy of the model was evaluated using the receiver operating characteristic (ROC) curve. The area under the curve (AUC) was calculated to quantify the model’s performance.

### Immune infiltration and functional analysis of URGScore subgroups

Immune infiltration and biological function differences between URGScore subgroups were evaluated using CIBERSORT with the LM22 signature matrix to assess the infiltration of 22 immune cell types^28^. Stacked bar plots and box plots were used to visualize immune cell differences between groups in the dataset. KEGG pathway activity scores between URGScore subgroups were compared using GSVA with the “limma” R package. KEGG pathway gene sets (c2.cp.kegg.v7.0.symbols) were downloaded from the MSigDB database for the GSVA analysis. GSEA was performed using the “clusterProfiler” R package to explore functional differences between high and low URGScore groups.

### Identification of URG-related signature genes

URG signature genes were identified from the DE-URGs using least absolute shrinkage and selection operator (LASSO) regression (glmnet package), support vector machine-recursive feature elimination (SVM-RFE; caret package), and random forest (RF; randomForest package). The GSE98320 dataset served as the training set, and the discriminative ability of the signature genes was validated using independent datasets GSE48581 and GSE50058.

### Subtype analysis of signature genes and URGScore

To evaluate the relationship between ubiquitination-related activity and specific rejection phenotypes, samples from the GSE98320 dataset were stratified into four groups based on the original study annotation: no rejection (*n* = 801), ABMR (*n* = 279), TCMR (*n* = 87), and mixed rejection (*n* = 41)^18^. The expression levels of the signature genes were compared among these groups. Pairwise statistical differences were assessed using the Wilcoxon rank-sum test (wilcox.test function in R).

To determine whether ubiquitination-related activity differed across rejection mechanisms, URGScore was calculated for all samples using ssGSEA implemented in the GSVA package (version 1.50.0). The discriminative performance of the URGScore was evaluated for two clinically relevant binary classifications: TCMR versus no rejection and ABMR versus no rejection. ROC curves and corresponding AUC values were generated using the pROC package in R.

### Protein–protein interaction (PPI) network

A PPI network was constructed for the signature genes using GeneMANIA^29^ (http://www.genemania.org) to identify proteins that potentially exhibit shared functions.

### Construction and validation of a diagnostic model

A signature URG-based diagnostic nomogram was constructed using the “rms” package (version 6.3-0). The model’s discriminative ability was assessed by AUC. Calibration was evaluated using a calibration curve. Calibration accuracy was quantified using the mean absolute calibration error, corresponding to the Integrated Calibration Index^30^. Decision curve analysis (DCA) was performed to estimate its potential clinical utility. The risk threshold was interpreted as the predicted probability of rejection at which a clinician might consider modifying immunosuppression or performing a confirmatory biopsy. In this framework, “treat all” and “treat none” represent interventions for all patients and none, respectively. Independent datasets GSE48581 and GSE50058 were used to validate the calibration and diagnostic performance of the model.

### Drug sensitivity prediction

The interaction between the signature genes and drugs was explored using the drug-gene interaction database (DGIdb, www.dgidb.org). Cytoscape software was employed to visualize the interaction network.

### Quantitative PCR validation of signature gene expression in renal allograft biopsies

To validate the expression of signature genes identified from transcriptomic analysis, quantitative PCR (qPCR) was performed using RNA isolated from 14 renal allograft biopsy samples (ID: 001–014). These samples were obtained from patients undergoing clinically indicated percutaneous renal biopsies due to acute graft dysfunction at Yantai Yuhuangding Hospital, Shandong, China. Biopsy indications included a sudden increase in serum creatinine exceeding 20% from baseline and/or new-onset or worsening proteinuria, particularly if > 3 g/24 h. Patients were excluded if they had blood clotting disorders, active infections, severe anemia, uncontrolled hypertension, heart failure, or were uncooperative with the biopsy procedure. The study was approved by the institutional ethics committee of Yantai Yuhuangding Hospital, and written informed consent was obtained from all participants. All methods were performed in accordance with the relevant guidelines and regulations, and all procedures involving human participants adhered to institutional and national ethical standards. The demographic and clinical characteristics of these patients are presented in Table 2.

**Table 2:** Clinical and demographic characteristics of renal allograft patients.

ID	Sex	Age	Transplant date	Pathological diagnosis	Creatinine (µmol/L)	Urea (mmol/L)
**001**	**M**	**62**	**2024-01-07**	**Acute TCMR (Banff IB), possible concomitant acute ABMR**	**258**	**13.86**
002	M	51	2023-03-15	Moderate chronic tubulointerstitial injury, possible CNI toxicity	305	20.68
003	M	52	2024-07-21	Acute tubular injury, likely ischemia-reperfusion	1006	28.10
**004**	**F**	**35**	**2024-08-03**	**Mixed acute rejection (TCMR IA + active ABMR)**	**228**	**14.48**
005	M	56	2018-11-11	Transplant IgA nephropathy	120	6.37
006	M	35	2018-04-25	Mild mesangial proliferative GN with segmental IgA deposits	129	3.92
007	M	40	2011-01-02	Ischemic injury with segmental MA-like lesions	379	19.53
008	F	56	2004-02-06	Diabetic glomerulosclerosis (type IV) with ischemic injury	190	14.80
009	M	46	2005-05-04	Focal proliferative IgA nephropathy with ischemic injury	208	16.33
010	F	55	2023-05-04	FSGS (cellular variant)	151	10.08
011	M	35	2024-11-12	TMA (transplant-associated)	535	31.60
012	M	36	2024-08-07	FSGS-like lesion	99	6.56
013	M	54	2011-06-09	IgA nephropathy with crescents and segmental sclerosis	199	12.98
014	M	28	2023-07-03	FSGS (NOS type), possible recurrence	129	10.20

Bolded samples denote mixed rejection, defined as biopsies exhibiting both TCMR and ABMR features.

*M* male, *F* female, *ABMR* antibody-mediated rejection, *CNI* calcineurin inhibitor, *FSGS* focal segmental glomerulosclerosis, *GN* glomerulonephritis, *MA* membranous-like appearance, *NOS* not otherwise specified, *TCMR* T cell-mediated rejection, *TMA* thrombotic microangiopathy

All patients were receiving standard triple immunosuppressive therapy (tacrolimus, mycophenolate mofetil, and prednisone). Pre-biopsy assessments included complete blood count, coagulation tests, liver and renal function, infection screening, and ultrasonography. Anticoagulants were withheld, and blood pressure was controlled prior to the procedure. Biopsies were performed under real-time Doppler ultrasound guidance using a 16G disposable automatic biopsy needle (Bard, USA) and local lidocaine anesthesia. Patients were placed in the supine position with a pillow under the back to elevate the transplanted kidney ventrally. The lower pole of the graft was targeted, and a puncture was performed at an oblique angle with a depth of 2–3 cm into the renal cortex. Patients were instructed to hold their breath during insertion to minimize movement. Three core tissue samples were retrieved per patient, each containing ≥ 10 glomeruli and ≥ 2 arterioles, pooled in liquid nitrogen, and mechanically homogenized together to obtain a single homogenized sample for RNA extraction. Following biopsy, the puncture site was compressed with gauze for 15–30 min. Patients were required to remain supine for 4–6 h with continuous monitoring of blood pressure, heart rate, and urine output. A follow-up ultrasound was performed after 12 h to evaluate for post-biopsy complications such as hematoma.

RNA was extracted using TRIzol reagent (Cat. #M00501; Suzhou Junxin Biotechnology, China) following mechanical disruption under cryogenic conditions. RNA quality and concentration were measured using a NanoDrop 2000c spectrophotometer (Thermo Fisher Scientific, USA). Residual genomic DNA was removed, and 1 µg of RNA was reverse-transcribed into cDNA using a reverse transcription kit (Cat. #CA01-100; Shenghanlun, China). qPCR was conducted using 2× SYBR Green qPCR Master Mix (Cat. #M00601; Shanghai Donghuan Biotechnology, China) on a CG-05 fluorescence qPCR system (Likang Biomedical, China). Primers targeting six genes *(DTX3L*,* MARCH1*,* NCF4*,* RNF125*,* TRIM21*,* TRIM22)* and the reference gene *GAPDH* were used. Each 20 µl reaction included 2 µl cDNA and was run in triplicate using standard three-step amplification. Relative gene expression levels were calculated using the 2^−ΔΔCt^ method. Primer sequences are provided in Table S1.

### Statistical analysis

All statistical analyses were conducted using R (v4.3.0). Expression data were log_2_-transformed during preprocessing to stabilize variance and improve distributional properties. Group comparisons were performed using Student’s *t* tests. Pearson correlation analysis was used to assess relationships between variables. A two-sided *p* value < 0.05 was considered statistically significant. Adjusted *p* values were used where multiple testing was performed.

## Results

### Identification and characterization of DE-URGs

**Fig. 1:**
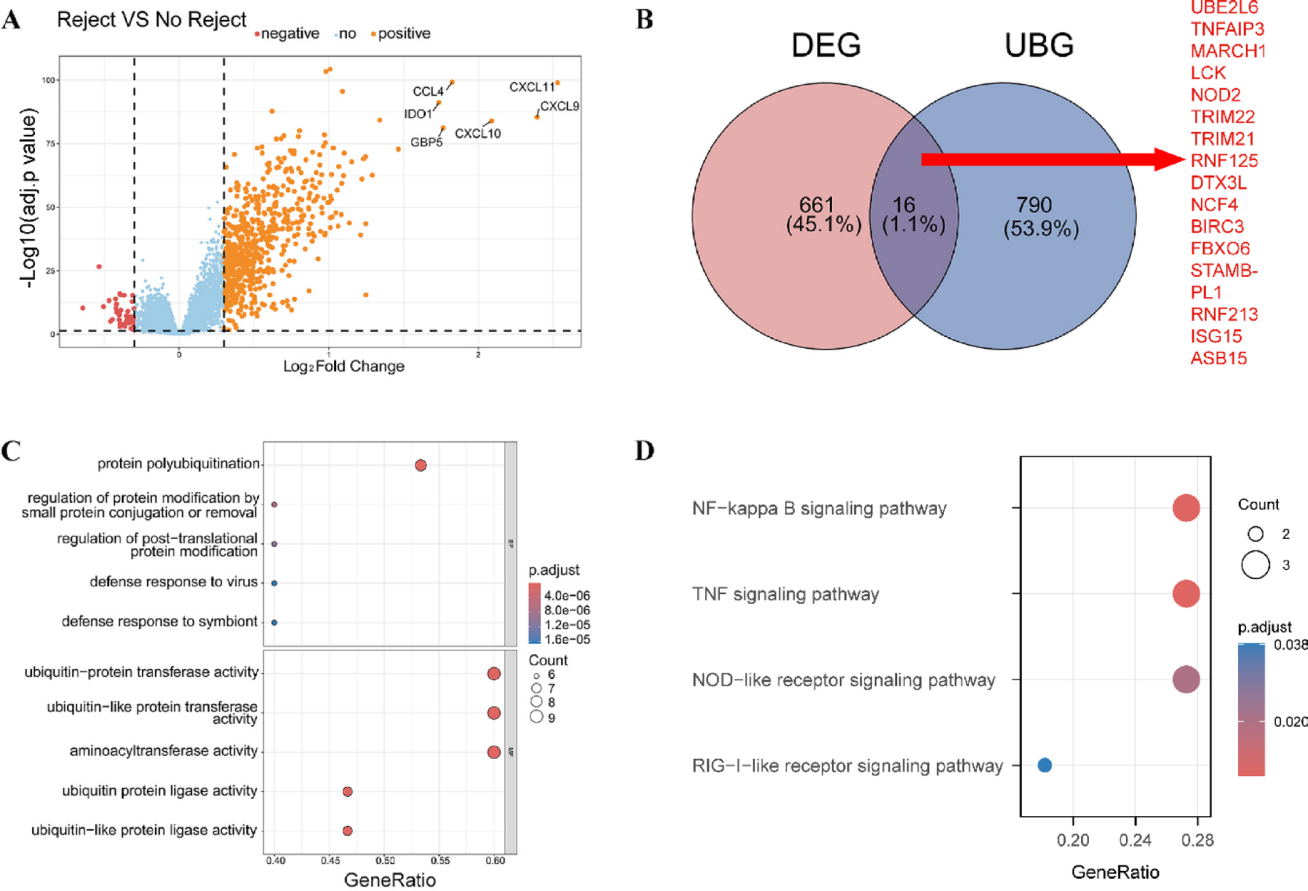
Differential expression and enrichment analysis of ubiquitination-related genes (URGs) in kidney transplantation rejection (KTR). (**A**) Differential expression analysis was performed on the GSE98320 dataset to identify genes associated with KTR. The volcano plot displays differentially expressed genes (DEGs) between rejection and no-rejection groups, with color indicating the direction of regulation: red for downregulated (negative), blue for non-significant, and orange for upregulated (positive) genes. Key upregulated genes were highlighted. (**B**) The Venn diagram shows the intersection between DEGs and URGs, identifying 16 ubiquitination-related genes (DE-URGs), including *ASB15*,* BIRC3*,* DTX3L*,* FBXO6*,* ISG15*,* LCK*,* MARCH1*,* NCF4*,* NOD2*,* RNF125*,* RNF213*,* STAMBP L1*,* TNFAIP3*,* TRIM21*,* TRIM22*,* and UBE2L6.* (**C**) GO enrichment analysis of DE-URGs indicates significant biological processes. (**D**) KEGG pathway enrichment analysis identifies key immune and inflammatory pathways involving the DE-URGs. Circle size indicates the number of genes involved in each pathway.

To identify KTR-related DEGs, we performed differential expression analysis on the GSE98320 dataset. A total of 677 DEGs were identified (Table S2, Fig. 1A), and intersecting these DEGs with 806 URGs yielded 16 DE-URGs (Fig. 1B), including *ASB15*,* BIRC3*,* DTX3L*,* FBXO6*,* ISG15*,* LCK*,* MARCH1*,* NCF4*,* NOD2*,* RNF125*,* RNF213*,* STAMBP L1*,* TNFAIP3*,* TRIM21*,* TRIM22*,* and UBE2L6*. To explore the biological significance of the DE-URGs, we conducted GO and KEGG enrichment analyses (Tables S3 and S4). In the GO analysis, the enriched biological processes included “protein polyubiquitination”, “regulation of protein modification by small protein conjugation or removal”, and “defense response to virus”. Regarding molecular functions, significant terms such as “ubiquitin-protein ligase activity” and “ubiquitin-like protein transferase activity” were enriched (Fig. 1C). KEGG pathway enrichment analysis highlighted the involvement of several key pathways, including the “NF-kappa B signaling pathway”, “TNF signaling pathway”, “NOD-like receptor signaling pathway”, and “RIG-I-like receptor signaling pathway” (Fig. 1D). These data indicate that URGs may play a critical role in modulating immune and inflammatory pathways, potentially influencing rejection mechanisms in KTR.

### URGScore model captures molecular characteristics linked to KTR

**Fig. 2:**
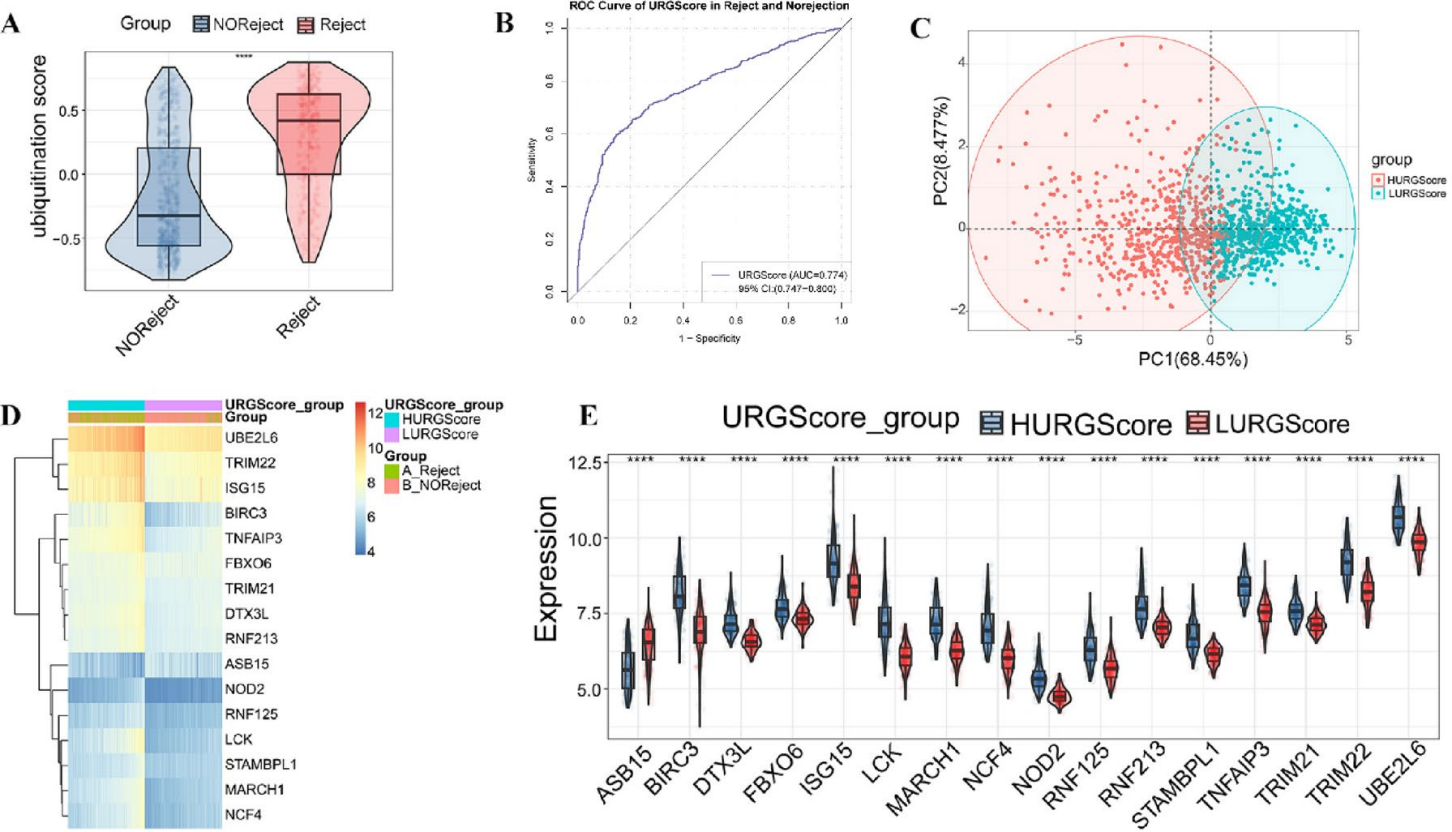
URGScore model construction and expression patterns of DE-URGs in KTR. (**A**) The URGScore was compared between the rejection group and the no-rejection group. (**B**) The ROC curve demonstrates the discriminative ability of the URGScore model. (**C**) The principal component analysis (PCA) reveals the distinction between high- and low-URGScore groups. (**D**) The heatmap illustrates distinct expression patterns of the DE-URGs between rejection and no-rejection groups. (**E**) The boxplots display significant differences in individual gene expression levels between the high- and low-URGScore groups. ****p < 0.0001.

To assess the expression patterns of DE-URGs and their association with KTR, we constructed a URGScore model based on 16 DE-URGs. The URGScore effectively distinguished between rejection and no-rejection groups, with significantly higher scores observed in rejection cases (*p* = 2.22 × 10^–16^; Fig. 2A). The model demonstrated good discriminative ability (AUC = 0.774, 95% CI 0.747–0.800) (Fig. 2B**)**, confirming its potential to differentiate patients based on rejection status. Additionally, a clear separation between high- and low-URGScore groups was observed in the PCA plot (Fig. 2C). The heatmap (Fig. 2D) further illustrated distinct expression patterns of the DE-URGs between the rejection and no-rejection groups, highlighting the upregulation of specific genes, such as *UBE2L6*,* TRIM22*, and *ISG15*, in rejection cases. Significant differences in DE-URG expression between high- and low-URGScore groups were also evident in the individual gene expression analyses (Fig. 2F). These data suggest that the URGScore effectively captures molecular characteristics linked to KTR.

### Immune and biological pathway differences between high- and low-URGScore groups in KTR

**Fig. 3:**
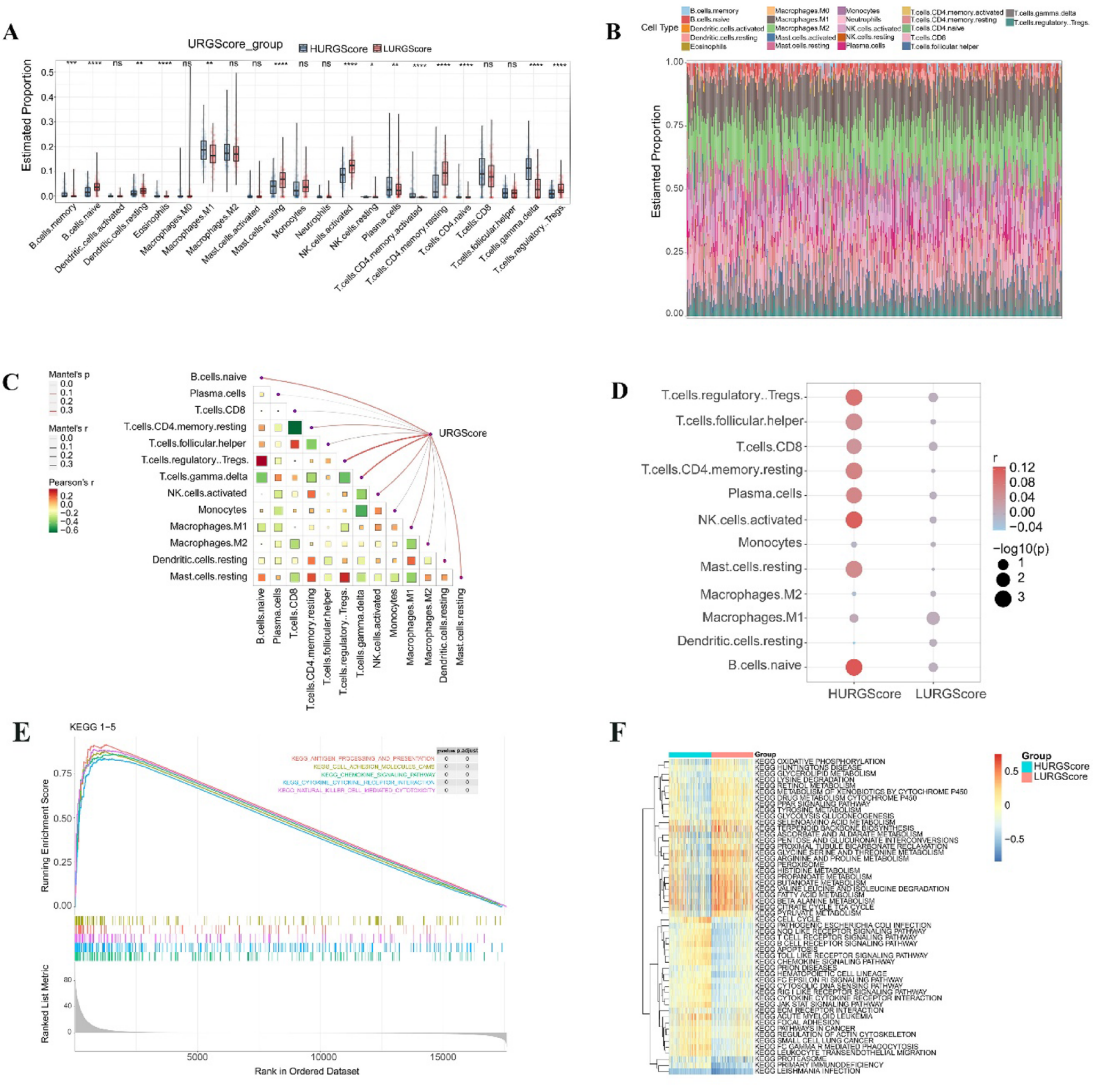
Immune cell infiltration and biological pathway analysis in high- and low-URGScore groups. (**A**) Differences in immune cell infiltration between the groups. (**B**) Immune cell composition in each sample. (**C**) The correlation between URGScore and various immune cell populations. The color gradient represents Pearson’s correlation coefficient (*r*), with red indicating a positive correlation and green indicating a negative correlation. The size of the squares represents the strength of Mantel’s test *p* value, with larger squares corresponding to stronger associations. (**D**) Immune cell correlations with high- and low-URGScore groups. The color gradient represents Pearson’s correlation coefficient (*r*), with red indicating a positive correlation and blue indicating a negative correlation. The size of the dots reflects the significance level, with larger dots corresponding to lower *p* values. (**E**) Activated immune-related pathways in the high-URGScore group. (**F**) Differential KEGG pathway enrichment between the groups.

Considering DE-URGs are closely involved in immune and inflammatory pathways, we sought to investigate the immune characteristics and biological pathways between high- and low-URGScore groups. Clear differences in immune cell distribution between the two groups (Fig. 3A) and the overall distribution of various immune cells within each sample (Fig. 3B) were observed. Regulatory T cells (Tregs), naive B cells, and gamma-delta (γδ) T cells showed positive correlations with URGScore (Fig. 3C), with Tregs having the strongest association in the high-URGScore group (Fig. 3D), suggesting their key role in the rejection process. Additionally, KEGG pathway analysis using GSEA (Table S5) revealed the activation of immune-related pathways in the high-URGScore group, such as antigen processing and presentation, cell adhesion molecules (CAMs), chemokine signaling pathway, cytokine–cytokine receptor interaction, and natural killer cell-mediated cytotoxicity pathways. (Fig. 3E). Consistent with these findings, GSVA (Table S6) further identified key immune pathways enriched in the high-URGScore group, including B cell receptor signaling, T cell receptor signaling, Toll-like receptor signaling, and NOD-like receptor signaling pathways (Fig. 3F). In contrast, metabolic pathways, such as lysine degradation, tyrosine metabolism, and histidine metabolism, were more prevalent in the low-URGScore group. These findings highlight the need to identify feature genes driving immune activity in the high-URGScore group to better understand and manage KTR.

### Identification of URG-related signature genes associated with KTR

**Fig. 4:**
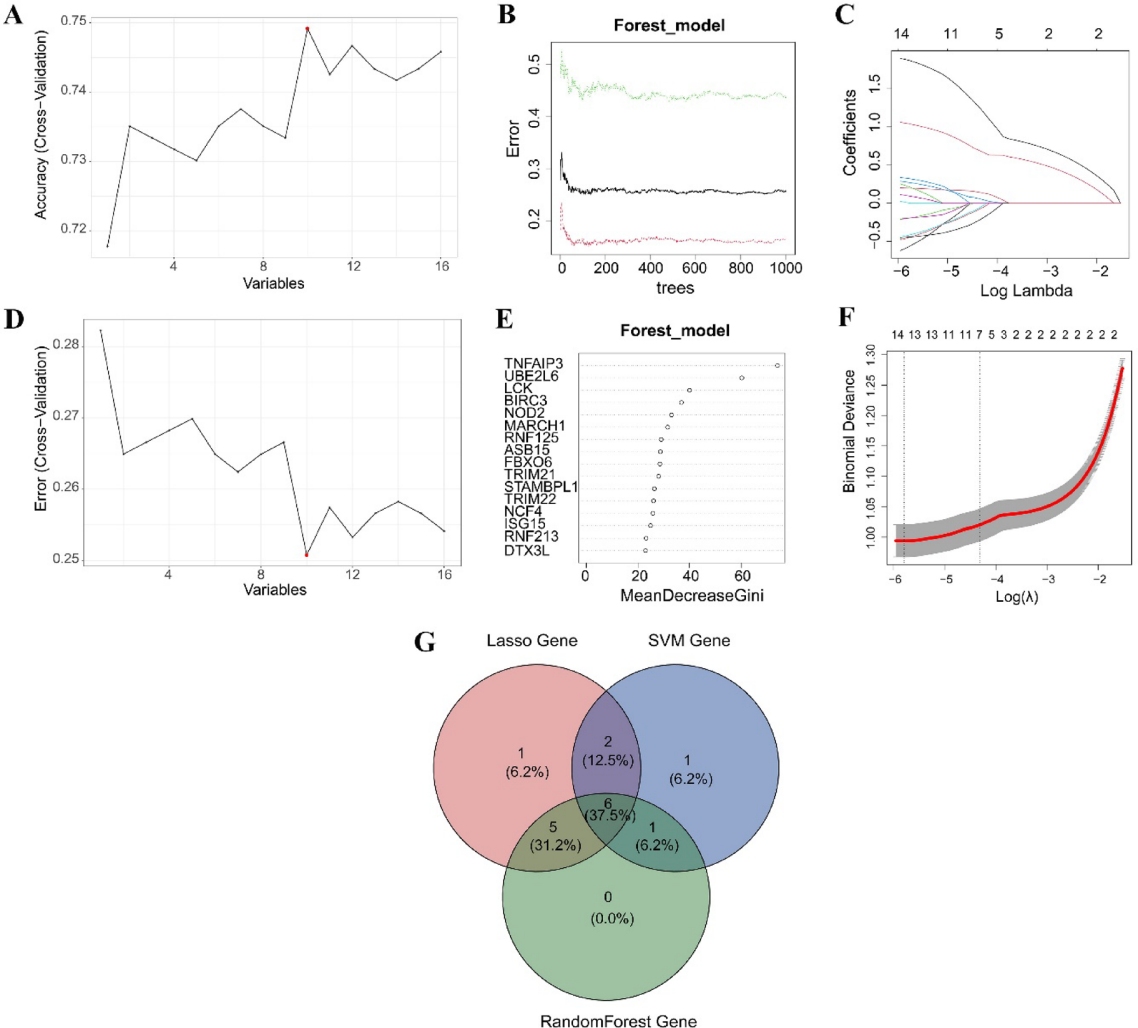
Machine learning analysis to identify signature URGs in KTR. (**A**, **D**) The SVM-RFE algorithm. Red points indicate the optimal variable sets. (**B**, **E**) The random forest (RF) model. The error plot and variable importance ranking display the results. (**C**, **F**) LASSO regression. The coefficients and binomial deviance are displayed across different log lambda values. (**G**) The intersection of genes identified by all three methods.

To identify URG-related signature genes linked to KTR, we applied three machine learning algorithms to the 16 DE-URGs. First, the SVM-RFE algorithm selected 10 genes based on accuracy and error rates across different variable sets (Fig. 4A and D). Next, the RF model identified 12 important genes by analyzing tree branches and using the mean decrease in Gini score to rank variable importance (Fig. 4B and E). Meanwhile, the LASSO regression method selected 14 genes by optimizing binomial deviance and cross-validation error (Fig. 4C and F). The intersection of genes identified by the three methods revealed 6 overlapping genes (*DTX3L*,* MARCH1*,* NCF4*,* RNF125*,* TRIM21*,* TRIM22*) as the most critical genes associated with KTR (Fig. 4G). To validate the findings, we compared the expression of the six signature genes between the rejection and non-rejection groups. Significant differences were observed in nearly all genes in the derivation set GSE98320 and validation datasets GSE48581 and GSE50058, suggesting their potential as reliable biomarkers for predicting rejection risk in KTR (Fig. S1A–C).

### The URG signature shows a graded increase across rejection subtypes and stronger discrimination of TCMR

**Fig. 5:**
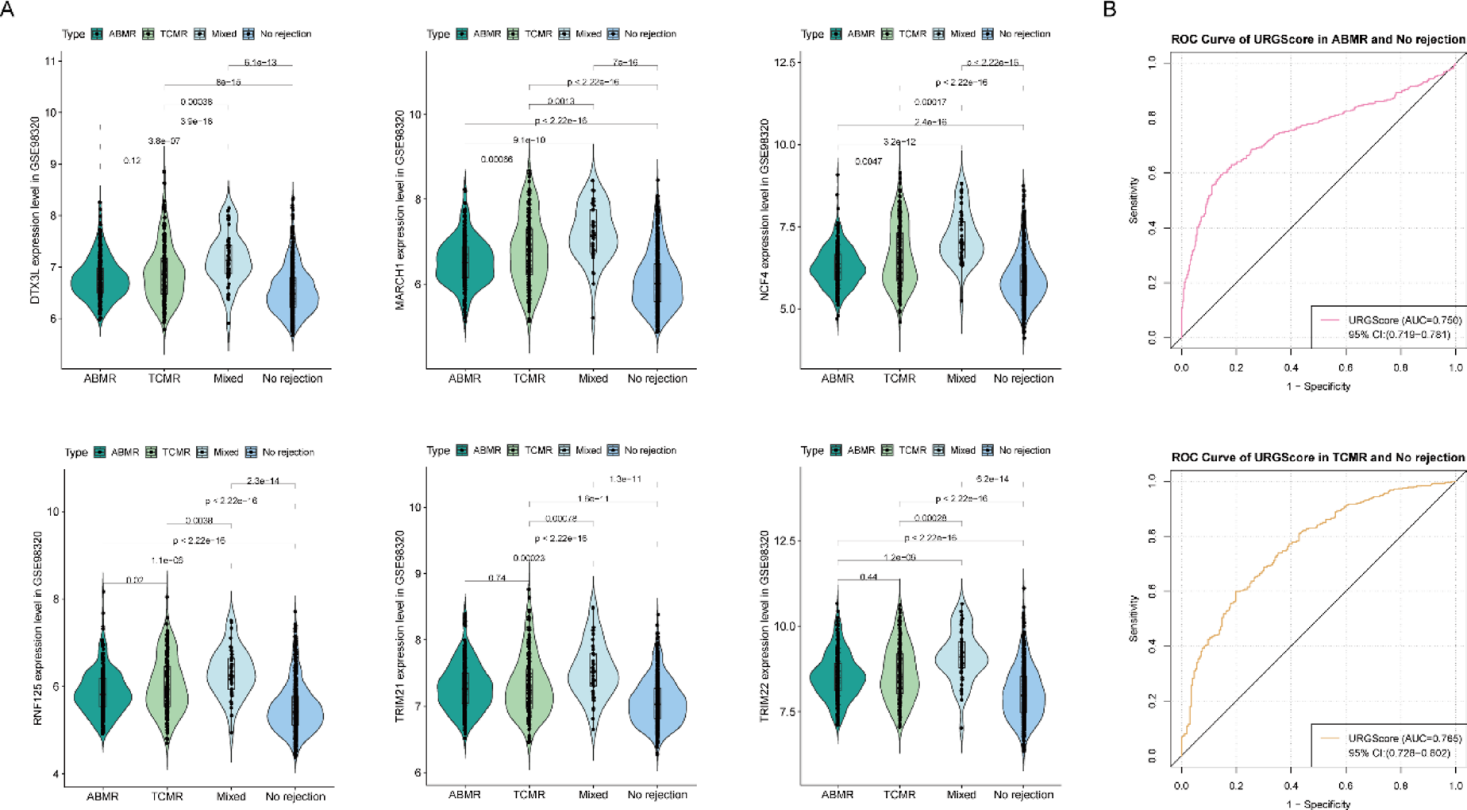
Subtype-dependent activation and diagnostic performance of the URG signature. (**A**) Violin plots showing the expression of the six signature genes across the four predefined groups in the GSE98320 dataset: no rejection, antibody-mediated rejection (ABMR), T-cell-mediated rejection (TCMR), and mixed rejection. (**B**) Receiver operating characteristic (ROC) curves depicting the performance of the URGScore in distinguishing ABMR from no rejection samples and TCMR from no rejection samples.

Having confirmed upregulation of the six URGs in rejection, variation in their expression across the predefined rejection subtypes in GSE98320 was assessed. All signature genes showed significant upregulation in each rejection subtype compared with the no rejection group. A significant graded increase in URG expression was observed, progressing from ABMR to TCMR and peaking in mixed rejection, a pattern consistent across all six genes and indicative of greater immunopathological complexity and T-cell-mediated inflammation (Fig. 5A).

The URGScore was then evaluated for its ability to distinguish rejection subtypes from no rejection samples. ROC analysis demonstrated strong discriminative ability for both ABMR (AUC = 0.750, 95% CI 0.719–0.781) and TCMR (AUC = 0.765, 95% CI 0.728–0.802), with greater sensitivity toward T-cell-mediated rejection (Fig. 5B). These findings indicate that the URG signature differentiates rejection from no rejection and reflects rejection subtypes, showing higher discriminative performance for TCMR.

### Development and validation of a nomogram based on six signature URGs for predicting rejection risk in KTR patients

**Fig. 6:**
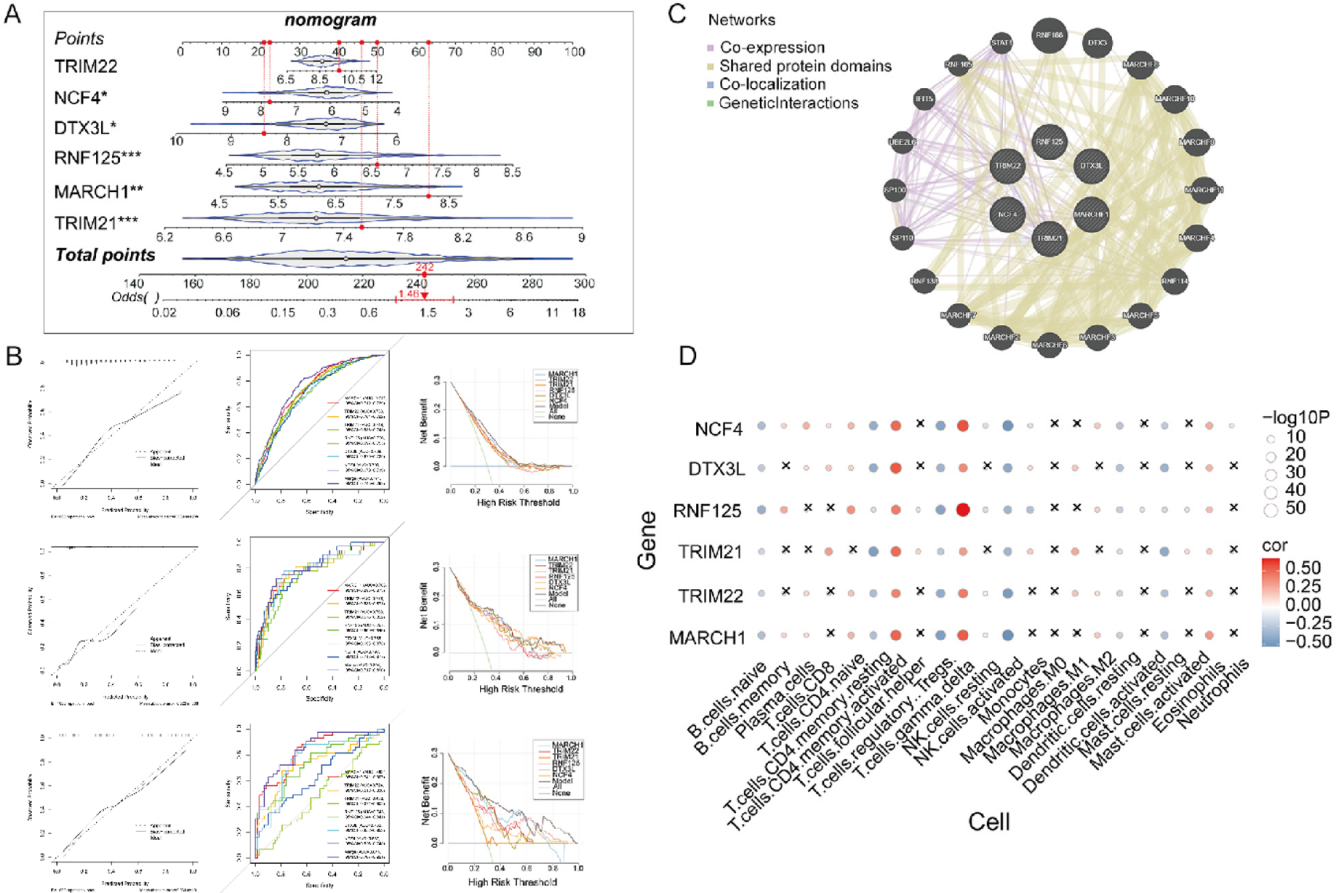
A nomogram based on six signature URGs for predicting rejection risk in KTR patients. (**A**) A nomogram was constructed based on the expression levels of signature URGs from the training dataset (GSE98320) to predict rejection risk in KTR patients. (**B**) Model performance was evaluated using calibration curves (left), ROC curves (middle), and decision curve analysis (DCA; right) in the training dataset (GSE98320; upper) and validated in the independent datasets GSE48581 (middle) and GSE50058 (lower). Mean absolute error, corresponding to the Integrated Calibration Index (ICI), is reported for the calibration curves. (**C**) A protein–protein interaction network was constructed. (**D**) Correlation analysis between the signature genes and various immune cell types. The color intensity represents the correlation strength, with red indicating positive correlations and blue indicating negative correlations. The size of the circles corresponds to the significance level, with larger circles representing stronger statistical significance.

Following the identification of six signature URGs, we constructed a nomogram using their expression levels from the training dataset (GSE98320) to predict rejection risk in KTR patients. The nomogram assigned points to each gene based on its expression, summing to a total score that correlates with the predicted risk (Fig. 6A). The performance of the model was evaluated using calibration curves, DCA, and ROC curves. In the training dataset (GSE98320), the calibration curve showed areas of miscalibration between predicted and observed rejection risk (Fig. 6B, upper left). ROC analysis showed that the model had an AUC of approximately 0.771 (95% CI: 0.745–0.798), which was higher than any single gene (Fig. 6B, upper middle). DCA indicated that the model significantly outperformed any individual URG for predicting KTR (Fig. 6B, upper right). Validation using independent datasets GSE48581 (Fig. 6B, middle) and GSE50058 (Fig. 6B, lower) produced consistent results. These data suggest that the nomogram based on six signature URGs provides promising discrimination for rejection risk in KTR patients.

To better understand the biological significance of the signature genes, we explored their potential interactions and functional relationships. The PPI network plot showed co-expression, shared protein domains, and genetic interactions among the genes, suggesting their involvement in complex regulatory pathways in KTR (Fig. 6C). Additionally, significant correlations were observed between the signature genes and various immune cell types, particularly with B and T cells, indicating a potential role in immune modulation during rejection (Fig. 6D). DGIdb annotation indicated that *NCF4* was associated with vincristine, rituximab, prednisone, idarubicin, doxorubicin hydrochloride, and cyclophosphamide (Fig. S2), suggesting possible therapeutic relevance.

### qPCR validation of the six signature URGs in independent renal allograft biopsy samples

**Fig. 7:**
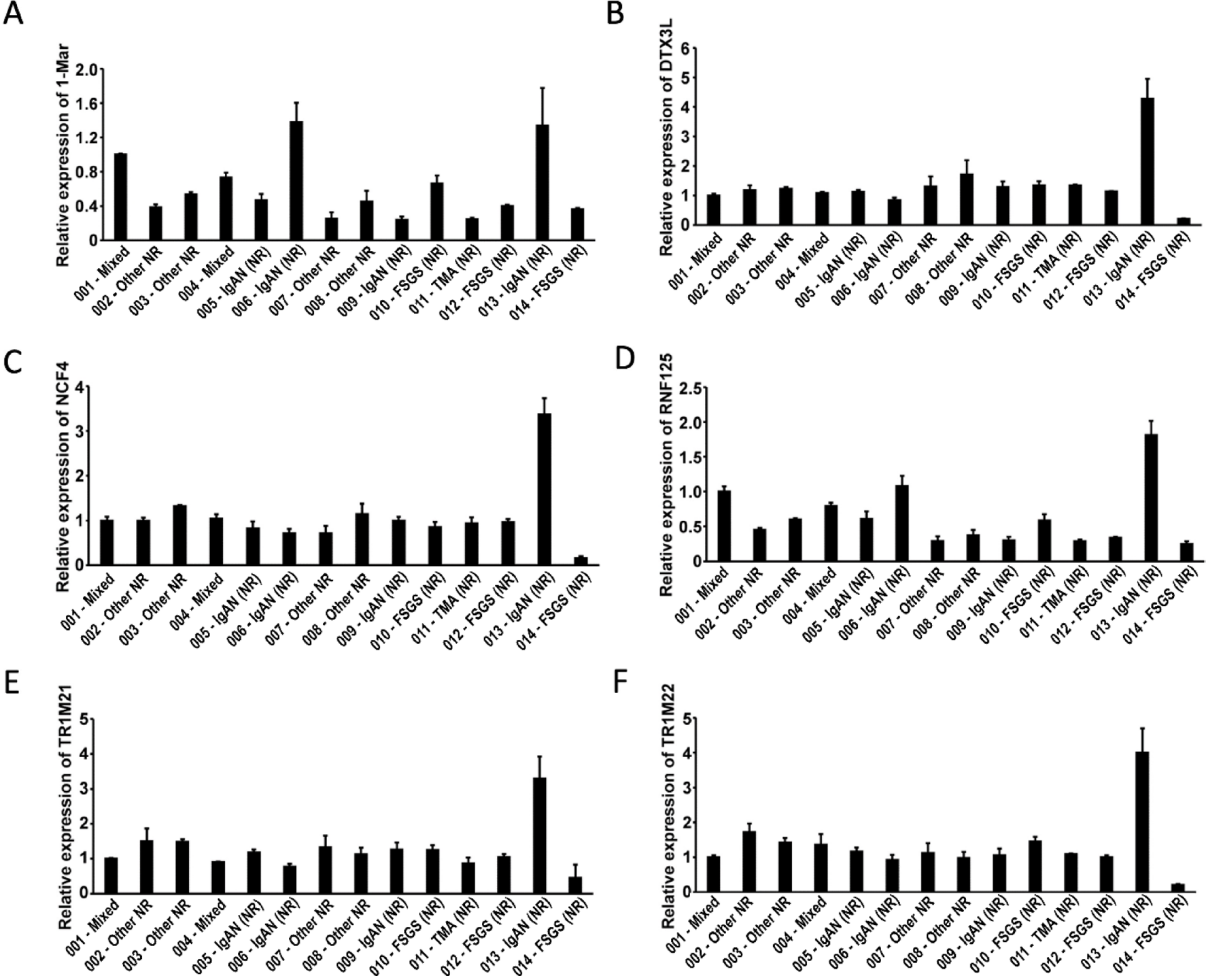
Validation of signature URG expression in kidney allograft biopsies using qPCR. (**A**–**F**) Relative mRNA expression levels of the six signature URGs, (**A**) *MARCH1*, (**B**) *DTX3L*, (**C**) *NCF4*, (**D**) *RNF125*, (**E**) *TRIM21*, and (**F**) *TRIM22*, were quantified by qPCR across 14 biopsy samples (001–014). Gene expression was normalized to GAPDH and presented as mean ± SD. Samples 001 and 004 represent mixed rejection (TCMR + ABMR). Sample 013 corresponds to a non-rejection case with IgA nephropathy with crescents and segmental sclerosis. ABMR: Antibody-mediated rejection; FSGS (NR): Focal segmental glomerulosclerosis, non-rejection; IgAN (NR): IgA nephropathy, non-rejection; Mixed: Mixed rejection (ABMR + TCMR); TCMR: T cell-mediated rejection; TMA (NR): Transplant-associated thrombotic microangiopathy, non-rejection; Other NR: other non-rejection pathologies (e.g., ischemic injury, diabetic glomerulosclerosis, calcineurin inhibitor toxicity).

To validate the transcriptomic findings, qPCR was performed on renal biopsy RNA samples from 14 kidney transplant recipients with various histopathological diagnoses. As shown in Fig. 7A–F, *MARCH1* and *RNF125* appeared higher in samples 001 and 004, both diagnosed with mixed rejection. Interestingly, sample 013, diagnosed as IgA nephropathy with crescents and segmental sclerosis, exhibited the highest expression of all six URGs. These findings suggest that URG upregulation may capture heightened immune activation before histological rejection is evident, supporting their utility in early detection of subclinical rejection or inflammation in KTR.

## Discussion

This study identified 16 critical genes associated with ubiquitination and immune response in KTR from transcriptomic datasets. A URGScore model was developed based on these DE-URGs, demonstrating significant discriminative accuracy for distinguishing between rejection and non-rejection cases. Six ubiquitination-related signature genes (*DTX3L*,* MARCH1*,* NCF4*,* RNF125*,* TRIM21*,* TRIM22*) were identified as potential predictors of KTR. Their expression showed a subtype-dependent gradient, increasing from ABMR to TCMR and reaching the highest levels in mixed rejection, indicating sensitivity to the intensity and complexity of alloimmune injury. In the qPCR cohort, *MARCH1* and *RNF125* showed higher expression in the two biopsies with a TCMR component, consistent with the transcriptomic patterns. Uniformly high expression of all six genes was observed in a biopsy diagnosed with crescents and segmental sclerosis, suggesting potential subclinical immune activation. These preliminary findings suggest that URG activation reflects intrarenal immune activity and may offer potential as early biomarkers for KTR, warranting further validation in larger cohorts.

The enrichment analyses of the DE-URGs revealed significant involvement in key immune pathways, as visualized in the KEGG pathway plot for different URGScore groups using GSVA analysis. Pathways related to ubiquitination mechanisms, such as NF-kappa B, TNF, and RIG-I-like receptor signaling, were enriched. These findings are consistent with the known roles of ubiquitination in immune regulation and transplant rejection^31^, where ubiquitination is activated via the ubiquitin-proteasome system to target damaged proteins for degradation, thereby modulating immune responses and maintaining protein homeostasis^32^. Enrichment analyses demonstrated the involvement of the NF-kappa B signaling pathway, which has been reported as significantly overexpressed in chronic ABMR cases, with *ICAM1* being a key overexpressed gene in this pathway​^33^. The TNF signaling pathway has also been implicated in KTR by promoting inflammation and apoptosis through activation of the NF-κB and MAPK pathways, leading to upregulation of pro-inflammatory cytokines and chemokines^34^. Furthermore, the RIG-I-like receptor signaling pathway, characterized by the upregulation of *RIG-I* and *IFIH1*, enhances immune activation in acute TCMR through NF-κB and IRF activation, driving cytokine production and immune cell recruitment^35^. These pathways are critically modulated by ubiquitination, which facilitates NF-κB activation through IκB degradation^36^, stabilizes TNF-mediated signaling complexes^37^, and supports TRIM25-mediated ubiquitination for RIG-I activation^38^, all contributing to immune responses during transplant rejection. Thus, our findings support the conclusion that ubiquitination processes play an active role in KTR, consistent with the mechanistic pathways identified in previous studies. The URGScore model effectively distinguished between rejection and non-rejection cases, highlighting the importance of these ubiquitination-related mechanisms.

A stratified analysis of rejection subtypes provides clearer insight into the biological role of the six-gene signature. The observed expression gradient, with mixed rejection > TCMR > ABMR > no rejection, is consistent with the known pathophysiology of these entities. The marked upregulation in ABMR suggests that the endothelial inflammation characteristic of humoral rejection is sufficient to activate ubiquitin-related pathways^39^. The substantially higher expression in TCMR indicates particular sensitivity of the signature to T-cell-driven processes, likely reflecting the dense interstitial T-cell infiltration and the IFN-γ-rich microenvironment that define this phenotype^40^. The peak expression in mixed rejection, in which humoral and cellular mechanisms are simultaneously active, suggests that the signature functions as a molecular rheostat that reflects the cumulative intensity and complexity of allograft injury. This dose-response pattern implies that the TCMR component is the principal contributor to signature activation. This interpretation helps explain the model’s good performance in predicting TCMR and provides a framework for understanding the qPCR findings, as both validated rejection samples contained a TCMR component. It also accounts for the high expression observed in the non-rejection biopsy with crescentic IgA nephropathy, a lesion known for pronounced T-cell infiltration and severe inflammation^41^. Therefore, the clinical value of the signature lies not in providing a specific diagnosis of rejection but in serving as a highly sensitive “danger signal” for severe intrarenal immune events driven by T cells.

Immune infiltration analysis revealed significant differences in immune cell populations between high- and low-URGScore groups, particularly an increase in Tregs and γδ T cells. This increase implies that during rejection, the immune system actively recruits these cells to the graft site. This is consistent with studies showing that Tregs are elevated during episodes of acute rejection as part of the body’s attempt to suppress excessive immune response^42^. Mechanistically, Tregs suppress immune responses by expressing CTLA-4 to downregulate co-stimulatory signals on antigen-presenting cells^43^ and secreting anti-inflammatory cytokines like IL-10 and TGF-β^44^. Increased Treg levels are linked to better graft outcomes and longer graft survival^45^. On the other hand, γδ T cells exhibit both pro-inflammatory and immunosuppressive functions. Studies have shown that γδ T cells can contribute to allograft rejection by producing IL-17^46^ and recruiting neutrophils^47^, which promotes inflammation and graft damage​. However, certain subsets of γδ T cells, particularly Vδ1 cells, have been associated with improved graft tolerance and better outcomes, likely through their ability to modulate immune responses and promote tissue repair^48^. The increase in γδ T cells observed in our URGScore analysis aligns with this dual role, suggesting that their recruitment may reflect an effort by the immune system to balance pro-inflammatory and regulatory mechanisms during rejection. Furthermore, ubiquitination may play a critical role in modulating the stability and activity of both FoxP3 in Tregs^49^ and key transcription factors in γδ T cells^50^, influencing their function and impact on graft survival. Regarding the six signature genes, each plays a distinct role in ubiquitination and immune regulation, with significant relevance to KTR. DTX3L (deltex E3 ubiquitin ligase 3) is an E3 ubiquitin ligase involved in antigen presentation and inflammatory signaling, and its upregulation in response to immune stimuli, such as IFN-α, may contribute to KTR by enhancing immune activation^51^. MARCH1 (membrane-associated ring-CH-type finger 1), another E3 ubiquitin ligase, modulates antigen presentation by regulating the ubiquitination and degradation of MHC class II and CD86 and is critical for controlling immune responses during transplant rejection^52^. An SNP (rs78140122) in *MARCH1* has been associated with acute rejection, further emphasizing its role in KTR^53^. *NCF4* (neutrophil cytosolic factor 4) regulates reactive oxygen species (ROS) production in immune cells^54^, and its upregulation in TCMR biopsies^55^ may exacerbate oxidative stress, promoting inflammation and contributing to KTR^56^. DGIdb annotations identified *NCF4* as a drug-associated gene. Given its role in regulating NADPH oxidase-mediated oxidative signaling, modulation of *NCF4* could theoretically influence pathways also targeted by current immunosuppressive therapies^57^. However, these implications remain speculative and require dedicated functional studies. RNF125 (ring finger protein 125) acts as a negative regulator of the RIG-I pathway and, along with Cbl-b, limits inflammasome activation by ubiquitinating NLRP3, which may help reduce excessive inflammation in KTR^58^. Finally, *TRIM21* and *TRIM22*, members of the *TRIM* (Tripartite motif) family, are involved in ubiquitination and proteolysis pathways during KTR, contributing to antigen processing, immune modulation, and exacerbating the immune response through IFN-γ-dependent mechanisms^59^. These genes highlight the complex interplay between ubiquitination and immune regulation in KTR. The nomogram developed from these signature genes showed potential utility for clinical risk assessment in KTR, and further external validation is needed. The DCA provides an estimate of possible clinical benefit, suggesting that the model may aid decisions such as when to adjust immunosuppression or consider a confirmatory biopsy. However, its practical deployment will require prospective studies to define clinically appropriate risk thresholds.

This study has several limitations. First, URG elevation reflects intrarenal immune activation, meaning that increased expression may also occur in other inflammatory conditions with prominent T-cell involvement, such as i-IFTA, crescentic glomerulopathies, infection-related tubulointerstitial injury, or polyomavirus nephropathy. Adjustment for these confounders was not feasible because the public datasets lacked detailed Banff scoring. Broader qPCR validation across these routine clinical lesions is necessary to define the signature’s specificity. Second, qPCR validation was limited by sample size (*n* = 14), including only two biopsy-proven rejection cases, due to the small number of patients undergoing biopsy at our institution. Future prospective studies with larger, multicenter cohorts are needed to validate the clinical utility of this nomogram. Notably, the high expression observed in a non-rejection case with IgA nephropathy suggests that the expression of these genes can vary depending on different pathological backgrounds or individual immune states. This highlights the importance of interpreting the model’s output within the patient’s specific clinical context. Third, the public datasets lacked uniform clinical parameters, such as serum creatinine, donor-specific antibodies status, and Banff lesion scores, which prevented direct comparison of the URGScore with standard-of-care models. Prospective cohorts with complete clinical annotation will be required to assess the incremental predictive value of the URGScore over existing diagnostic approaches. In addition, although ROC and DCA suggested improved discrimination and net benefit of the combined signature compared with individual markers, formal statistical tests for incremental predictive value (such as DeLong tests, NRI, or IDI) were not performed and should be considered in future studies. Fourth, our study is correlational. Future functional studies using cell culture or animal models are essential to elucidate the precise mechanistic roles of these URGs in the pathophysiology of rejection and to validate their potential as therapeutic targets. Lastly, the training set calibration showed clear miscalibration. A fully unbiased internal validation would require rerunning the entire feature-selection pipeline within each bootstrap, which should be addressed in future work.

In conclusion, this study identified six signature URGs associated with KTR and developed a nomogram that shows potential for predicting rejection risk. Functional enrichment analysis revealed the involvement of immune and inflammatory pathways, emphasizing the role of ubiquitination in immune modulation during rejection. In the qPCR cohort, *MARCH1* and *RNF125* showed higher expression in the two biopsies with a TCMR component, and uniformly elevated expression of all six URGs was observed in a crescentic IgA nephropathy biopsy, suggesting that these genes may reflect broader intrarenal immune activation. Together, these findings provide a potential tool for early KTR risk stratification while offering new molecular insights into ubiquitination-mediated immune regulation in allograft rejection.

## Supplementary Information

Below is the link to the electronic supplementary material.

Supplementary Material 1

Supplementary Material 2

Supplementary Material 3

Supplementary Material 4

Supplementary Material 5

Supplementary Material 6

Supplementary Material 7

Supplementary Material 8

Supplementary Material 9

## Data Availability

All data generated or analysed during this study are included in this published article and its Supplementary Information files.
