# Supplementary figures and images for: Identification of ubiquitination-related signature genes for predicting kidney transplant rejection
Source: Sci Rep. 2026 Feb 10;16:8102. doi: 10.1038/s41598-026-38022-8 (PMC12961045; doi:10.1038/s41598-026-38022-8)

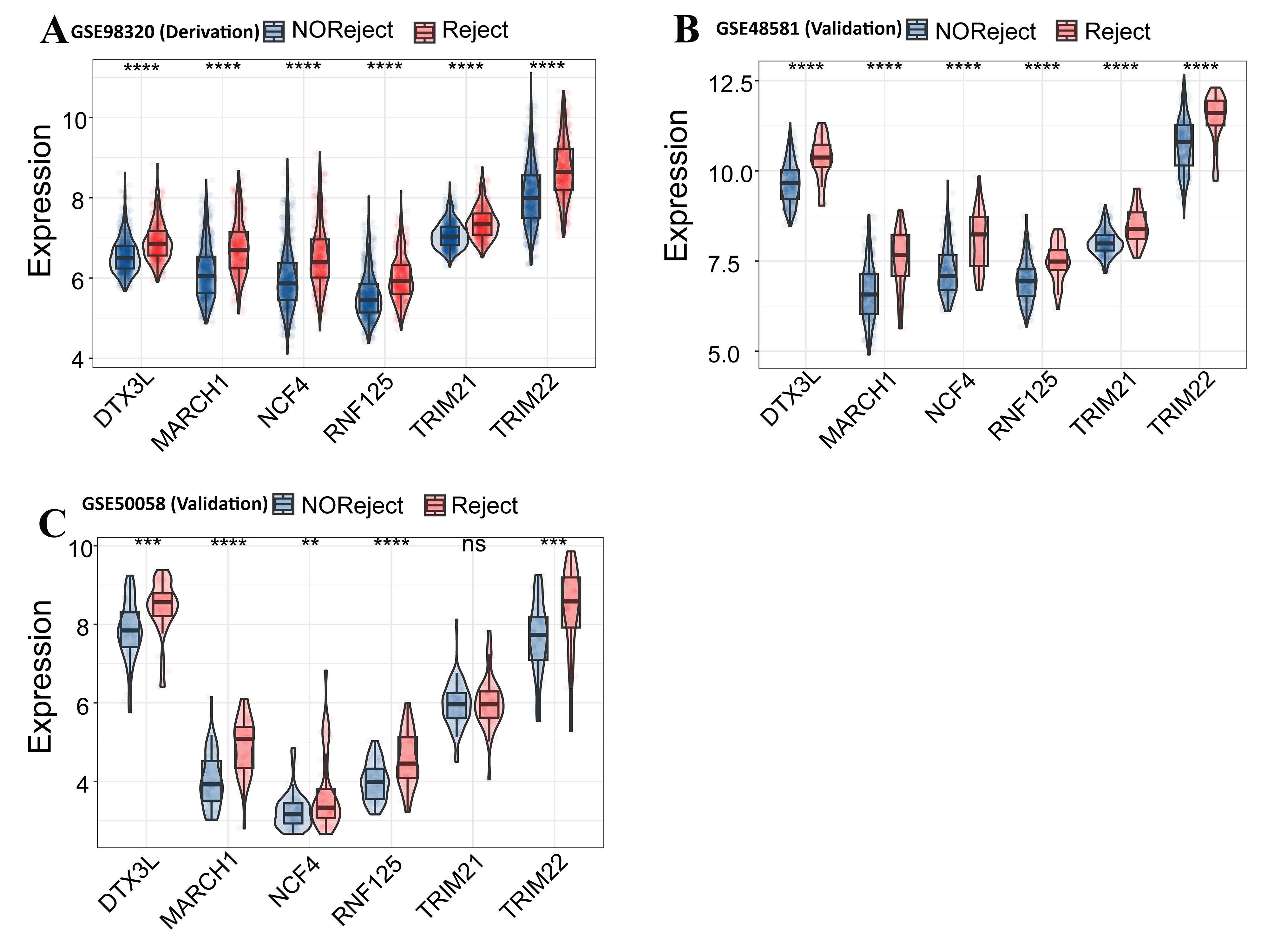

Supplement: Supplementary file 1 — Supplementary Material 1 [file 41598_2026_38022_MOESM1_ESM.jpg]

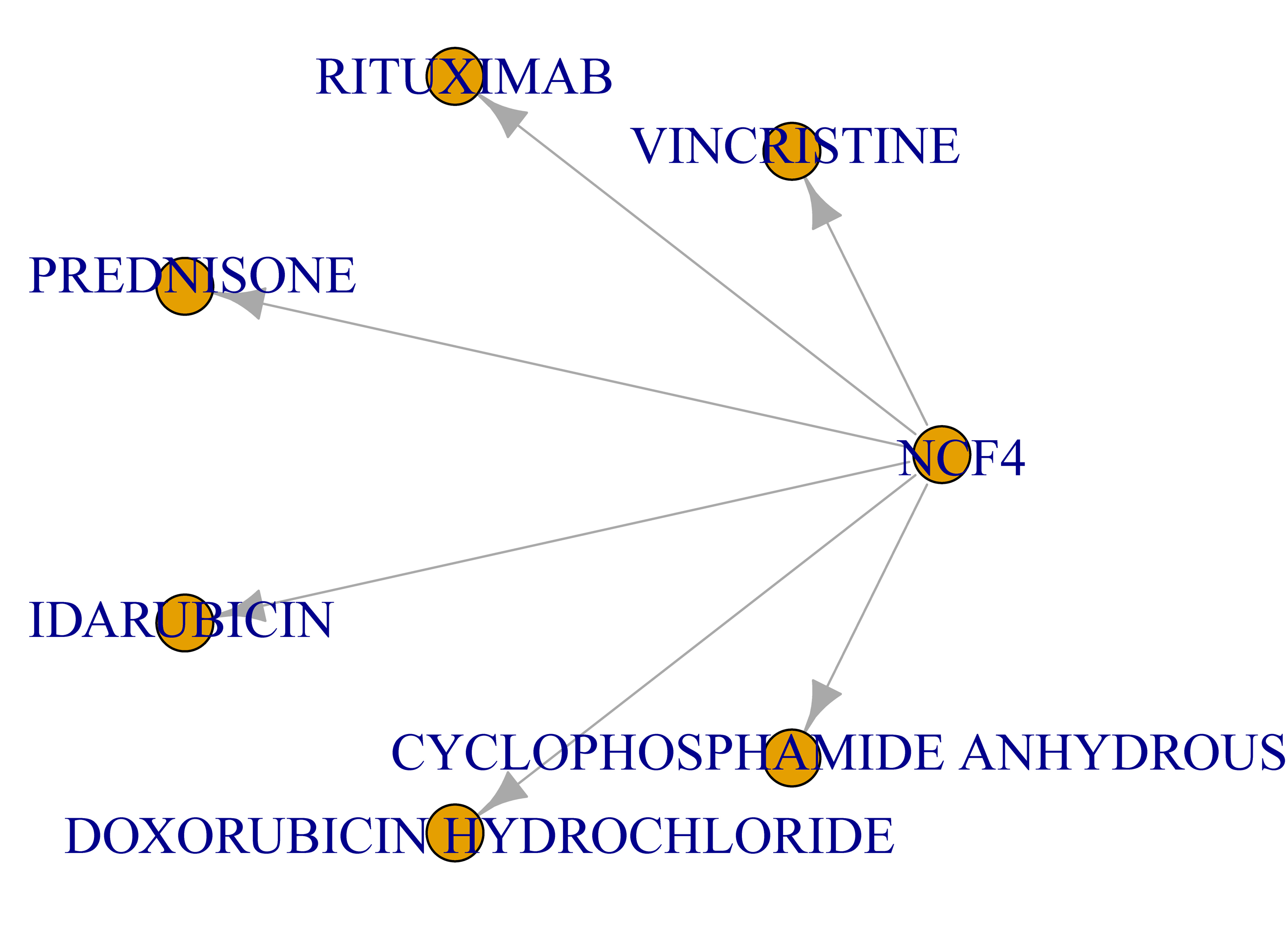

Supplement: Supplementary file 2 — Supplementary Material 2 [file 41598_2026_38022_MOESM2_ESM.jpg]
