# Supplementary material for: Identification of ubiquitination-related signature genes for predicting kidney transplant rejection
Source: Sci Rep. 2026 Feb 10;16:8102. doi: 10.1038/s41598-026-38022-8 (PMC12961045; doi:10.1038/s41598-026-38022-8)
Supplement: Supplementary file 9 — Supplementary Material 9 [file 41598_2026_38022_MOESM9_ESM.docx]

**Supplementary Figure S1. Expression of signature genes between rejection and non-rejection groups in KTR across three datasets.** (A–C) The expression of signature genes was compared between the rejection and non-rejection groups in GSE98320 (derivation), GSE48581 (validation), and GSE50058 (validation), respectively. ^*^*p* < 0.05, ^***^*p* < 0.001, ^****^*p* < 0.0001, ns: not significant.

**Supplementary Figure S2. Drug-gene associations predicted for *NCF4*.** Seven candidate compounds associated with *NCF4* were identified using DSigDB enrichment analysis. Nodes represent *NCF4* and the associated compounds; edges indicate predicted drug-gene associations.
